# Erratum for Euro Surveill. 2018;23(31)

**DOI:** 10.2807/1560-7917.ES.2018.23.33.1808161

**Published:** 2018-08-16

**Authors:** Eurosurveillance editorial team

**Affiliations:** 1 European Centre for Disease Prevention and Control (ECDC), Stockholm, Sweden

In the article ‘Epidemiology and resistance phenotypes of carbapenemase-producing Klebsiella pneumoniae in Greece, 2014 to 2016’ published on 02 August 2018, ≤ 32mg/L was erroneously reported as ≤ 2mg/L. The mistake was corrected on 10 August 2018. 

